# Medication Based Recovery in Opioid Use Disorder: A Clinical and Epistemic Imperative

**DOI:** 10.7759/cureus.99631

**Published:** 2025-12-19

**Authors:** Arya Babul, Parisa Mahdavi, Momina Hussain, Zaigham H Janjua, Najib Babul

**Affiliations:** 1 Biomedical Sciences, West Career and Technical Academy, and Society for Awareness of Tropical Diseases, Neveda, USA; 2 Drug Development, Tavolar LLC, Las Vegas, USA; 3 Genomics, Chinese Academy of Tropical Agriculture Sciences, Sanya, CHN; 4 General Medicine, Dr Akbar Niazi Teaching Hospital, Islamabad, PAK; 5 Drug Development, Cinergen LLC, Las Vegas, USA

**Keywords:** epistemic indifference, epistemic injustice, harm reduction, medication‑based recovery, medication for opioid use disorder, opioid use disorder, policy and payment reform, polysubstance abuse, retention in care, stigma reduction

## Abstract

Medication‑based recovery (MBR) is based on a recent proposal by leading addiction scientists, which rejects abstinence‑only definitions that drive unsafe practices such as forced tapering or discharge. Instead, it reframes opioid use disorder (OUD) treatment by recognizing that recovery can be achieved and sustained while patients remain on medication for OUD (MOUD). MBR is structured around a staged care pathway of protection, remission, and recovery, prioritizing immediate harm reduction, sustained clinical engagement, and measurable improvements in health and social functioning. It endorses ongoing MOUD combined with validated monitoring, behavioral interventions, and social supports to reduce overdose, infectious complications, and other harms.

Effective implementation of MBR requires an aligned policy and payment reforms to support measurement‑based practice, coordinated services and integrated delivery, licensing and credentialing changes to protect clinicians who provide evidence‑based MOUD, and workforce development to expand prescribers and embed addiction expertise in primary care and telehealth. MBR also requires public and family education to reduce stigma and to reframe expectations about what constitutes meaningful recovery. Success should be measured by retention on MOUD and reductions in overdose risk during the protection phase, decreases in use and symptom severity during remission, and gains in housing, employment, and social functioning during recovery.

Framed as both a clinical and an epistemic imperative, MBR affirms the legitimacy of evidence‑based care and counters epistemic exclusion that marginalizes patients and clinicians who rely on MOUD. Widespread adoption of MBR holds the promise of reducing mortality, improving health, and expanding equitable recovery opportunities for patients and families.

## Editorial

Introduction: reframing recovery

The good physician treats the disease; the great physician treats the patient who has the disease. - Sir William Osler, Aequanimitas (1899)

The best ideas transform practice by clarifying what clinicians should measure, what systems should fund, and what families and patients can reasonably expect. We commend McLellan and Volkow for advancing a deceptively simple yet profoundly transformative idea: medication‑based recovery (MBR), as presented in a recent issue of the New England Journal of Medicine [1]. They emphasize that recovery is both achievable and worthwhile while a patient remains on medication for opioid use disorder (MOUD), challenging the long‑standing conflation of recovery with abstinence. By asserting that “abstinence is neither necessary nor sufficient for recovery,” they reshape the narrative and bring it closer to clinical reality. Though brief, their comment carries an outsized impact. It is not merely a semantic shift but a strategic recalibration with far‑reaching implications for policy, practice, stigma, and outcomes. It redirects clinical priorities toward saving lives, improving function, and ensuring that regulatory and reimbursement incentives align with contemporary addiction science.

This reframing does not occur in isolation. For decades, addiction treatment in the United States has been dominated by abstinence‑only philosophies, often rooted in moral or punitive traditions rather than medical science. While such approaches have benefited some, they have also excluded many patients from care, reinforced stigma, and perpetuated cycles of relapse and overdose. MBR represents the next stage in this evolution: a model that acknowledges opioid use disorder as a chronic condition requiring sustained therapeutic engagement, much like diabetes or hypertension. By situating recovery within a chronic‑care framework, MBR affirms that ongoing treatment is not a failure but a legitimate pathway to health and dignity.

Scientific authority

Thomas McLellan has long been recognized as a leading addiction researcher and policy expert whose work has shaped our understanding of substance use disorders. Equally influential is Nora Volkow, Director of the National Institute on Drug Abuse (NIDA), whose neuroimaging research reframed substance use disorders as chronic medical conditions rather than moral failings. Together, their scientific authority lends substantial weight to the concept of MBR and strengthens the call to align policy and practice with modern addiction science.

The impact of their work is amplified by decades of foundational contributions. McLellan’s research on treatment outcomes and the Addiction Severity Index provided the field with standardized tools to measure progress, while Volkow’s neuroimaging studies, and more recently her stewardship of NIDA, have elevated addiction research to new heights [2,3]. Both have consistently advanced the principle that evidence, not ideology, should guide treatment. Their endorsement of MBR, therefore, carries not only scientific weight but also symbolic power, signaling to policymakers and clinicians that the time has come to normalize MBR as mainstream practice.

Clinical realism and patient safety

MBR is important first and foremost because it anchors care in clinical realism and patient safety. The staged approach begins with rapid initiation of MOUD to prevent withdrawal, overdose, and infectious complications, affirming that immediate harm reduction is both humane and evidence‑based. Early protection establishes the physiological and social supports that enable patients to pursue remission and, ultimately, broader recovery.

In clinical practice, MOUD should not be viewed as a temporary bridge or an endpoint but as an enduring system. Under optimal dosing plans, combined with behavioral interventions and social services, patients can achieve sustained reductions in opioid misuse and measurable improvements in health and social functioning, often while remaining on medication.

The stakes are high as untreated OUD is associated with staggering mortality, infectious disease transmission, maternal morbidity, and profound social disruption [4]. Rapid initiation of MOUD is therefore not simply a clinical option but a moral imperative. Just as insulin minimizes diabetic complications and antiretroviral therapy prevents HIV progression, MOUD prevents overdose and stabilizes physiology. Clinical realism demands that we treat OUD with the same urgency and pragmatism as other chronic conditions, recognizing that safety and stabilization are prerequisites for long‑term recovery.

Avoiding counterproductive practices

MBR reduces counterproductive decisions that persist in many programs. The threat of discontinuing life‑saving medications when patients fail to meet arbitrary abstinence criteria is both risky and countertherapeutic. The staged model instead advocates for ongoing MOUD provision with intensified clinical supports rather than medication cessation and discharge. For clinicians, this requires a different posture at the first visit, when instead of offering a fixed taper or an uncertain timeline to abstinence, they should present a staged pathway comprising immediate protection, targeted efforts toward remission, and broader recovery objectives, with MOUD available as a durable support throughout that trajectory.

Detoxification‑only programs illustrate the danger of these outdated practices. Relapse rates often exceed 80% within months, underscoring the futility of short‑term abstinence mandates. By contrast, sustained MOUD dramatically reduces overdose risk and improves retention in care. The staged model, therefore, represents not merely a philosophical shift but a practical safeguard against avoidable harm. Clinicians who adopt this approach can reassure patients that recovery is measured by improvements in health and function rather than adherence to arbitrary timelines.

Measurement, monitoring, and linkages

Operationalizing MBR at the patient level requires routine, non‑punitive clinical monitoring. Outdated drug‑testing schemas that function as surveillance rather than support should be replaced with validated measures of substance use, physical and mental health, and social functioning. Monitoring must be framed as supportive rather than punitive, so patients experience it as a tool for tailoring care rather than a mechanism of control.

Validated instruments already provide structured ways to assess progress [5-7]. Emerging AI‑enabled tools for clinical monitoring and risk prediction can further strengthen this framework, provided their use is transparent, ethical, and aligned with evidence‑based care. When integrated thoughtfully, such innovations allow clinicians to track progress, adjust care plans in real time, and avoid arbitrary thresholds that too often lead to premature discharge.

Equally urgent is the need for office‑based prescribers who do not provide holistic services to establish clear referral pathways to behavioral health, social support, and specialty addiction programs. The staged model anticipates these linkages, ensuring that patients move beyond protection toward remission and recovery through coordinated, multidisciplinary care.

Policy and payment reform

Policy and payment reforms are essential to scaling MBR; however, many insurers continue to inadequately cover routine monitoring, counseling, and care coordination for medications for opioid use disorder (MOUD), services that are critical to successful implementation of this model [8,9]. Payment structures must evolve to support measurement‑based care, case management, and timely behavioral interventions so clinicians can provide comprehensive recovery pathways rather than episodic detoxification. Licensing and reimbursement standards that distinguish medication‑offering programs from abstinence‑only programs are anachronistic, impeding integrated care and reinforcing clinician fears of sanction.

Underlying this rigidity are definitions of recovery that exclude MOUD, reflecting not only clinical misalignment but also epistemic exclusion, sidelining both scientific evidence and lived experience. Regulatory frameworks should mandate that licensed programs make evidence‑based tools, including MOUD, available or facilitate seamless referrals between sites and programs when needed. Clinicians implementing MBR must also be shielded from punitive regulatory or credentialing responses premised on outdated definitions of recovery. No practitioner should face professional risk for offering evidence‑based care that ensures safety, engagement, and long‑term healing.

Payment reform can take several forms, such as bundled reimbursement for MOUD plus counseling, parity enforcement to guarantee addiction care coverage equivalent to other chronic diseases, and expanded telehealth reimbursement to reach rural and underserved populations. Regulatory frameworks should also incentivize integrated care models that combine medical, behavioral, and social supports. Without such reforms, clinicians remain trapped in outdated reimbursement structures that reward episodic detoxification rather than sustained recovery. Aligning payment with evidence‑based practice is therefore not optional but imperative to scaling MBR.

Stigma, families, and education

Stigma and family expectations remain practical barriers to acceptance of MBR. Many families and communities continue to equate “recovery” with complete abstinence, while MBR reframes recovery around meaningful improvements in health, safety, and functioning. Public communication that normalizes medication‑assisted approaches, similar to the way chronic diseases are treated with continuous therapy, can reduce stigma and better align expectations with clinical realities [10].

Clinicians, too, may carry implicit biases or outdated training that equates abstinence with success [11,12]. Systems and providers should proactively offer clear, accessible education for families and care teams, explaining the cascade (protection → remission → recovery), why MOUD may be lifelong for some patients, and how progress will be tracked and supported. Public education campaigns can draw lessons from HIV/AIDS advocacy, which successfully reframed a stigmatized condition into one understood as treatable and manageable.

Families must be guided to see MOUD not as “substituting one drug for another” but as stabilizing therapy that restores safety and function. Clinicians require ongoing training to unlearn biases and to embrace recovery definitions that prioritize dignity and well-being. By normalizing medication‑based recovery, systems can foster a culture in which patients are supported rather than judged, and recovery is measured by health, function, and quality of life rather than abstinence alone [13].

Scaling and workforce development

A limited number of patients at risk of overdose currently receive MOUD, and many are treated only with short detoxifications rather than long‑term care. Scaling MBR requires training more prescribers, embedding addiction expertise in primary care, and expanding telehealth and mobile services to reach underserved communities [14].

MBR is not a one‑size‑fits‑all prescription. Some patients present with polysubstance use, co‑occurring disorders, or social needs that necessitate prolonged, multidisciplinary services. The model does not promise a quick or easy cure; instead, it requires sustained engagement and service intensification when necessary.

Workforce shortages remain a critical barrier, with fewer than half of patients with opioid use disorder currently receiving MOUD. Expanding access demands interdisciplinary training that equips primary care, emergency medicine, and psychiatry providers to deliver evidence‑based treatment [15]. Scaling MBR is therefore not simply a matter of prescribing more medication; it requires systemic investment in people, infrastructure, and technology to ensure that recovery is accessible, durable, and equitable.

Defining success in recovery

Success must be measured according to the cascade’s logic and individual patient needs. Protection is represented by retention on MOUD and reductions in overdose risk; remission corresponds to decreases in use and symptom severity; and recovery is characterized by improvements in health, housing or job stability, and social functioning. The temporal benchmarks McLellan and Volkow describe (early: 1-6 months; sustained: 7-12 months; stable: >12 months) provide a practical guide for clinics and systems [1].

Metrics must move beyond the binary abstinence‑versus‑use model that neglects meaningful progress. Composite outcomes that capture safety, function, and quality of life yield a more accurate portrayal of recovery [16]. Equally important are patient‑centered outcomes, such as restored family relationships, community participation, and self‑reported well-being, that often matter more to patients than abstinence alone and that reflect the broader social determinants of health. By adopting these measures, systems can capture the full spectrum of recovery and avoid reductive metrics that obscure progress.

Responding to criticisms

Common objections to MBR deserve direct responses. Critics who argue that MOUD “perpetuates dependency” misunderstand the clinical analogy: treating opioid use disorder as a chronic medical condition and utilizing MOUD for long‑term therapy is analogous to accepted treatments for countless chronic conditions, where ongoing medication is expected and beneficial. Equating recovery only with abstinence runs counter to empirical definitions that emphasize reduced problematic use and improved well-being. For many, MOUD facilitates recovery by stabilizing neurobiology and behavior, making psychosocial benefits possible. Cost concerns likewise overlook the fiscal and human toll of untreated OUD, overdoses, infectious disease, criminal justice involvement, and lost productivity, which far exceed the cost of sustained MOUD plus supportive services.

Clinicians are trained to expect adherence, and when patients struggle to follow instructions, whether in diabetes, hypertension, or OUD, frustration is understandable. In addiction care, however, such frustration too often evolves into moral disapproval, particularly toward socially marginalized patients. MBR calls for a recalibration in emotional posture where patients who falter require supplemental support, not withdrawal of care. Tempering the impulse to respond punitively to adherence problems is not apologism but clinical realism, grounded in a growing body of evidence demonstrating that MOUD saves lives even when progress is slow or nonlinear.

The culture that states MOUD “substitutes one drug for another” ignores the distinction between chaotic, compulsive use and medically supervised therapy that restores function. Addressing these criticisms directly is essential to building a consensus that medication-based recovery is therapy rather than dependency, and evidence-based care rather than indulgence.

Conclusion: clinical and epistemic imperative

Adopting MBR will require clinician training, payment reform, regulatory realignment, and a reframing of recovery expectations. The imperative is clear that when a treatment reduces mortality and promotes functional recovery, systems should ensure its availability, and clinicians should use it as a bridge to fuller recovery rather than a barrier. Definitions of recovery that exclude MOUD reflect not only clinical misalignment but epistemic exclusion, sidelining both scientific evidence and lived experience.

Medication‑based recovery is therefore both a clinical and an ethical imperative. Recognizing recovery through MOUD affirms the dignity of patients, the integrity of medicine, and the responsibility of systems to align practice with evidence.
